# First documentation of *Aspergillus welwitschiae* in COVID-19-associated pulmonary aspergillosis in the Americas

**DOI:** 10.1590/S1678-9946202567008

**Published:** 2025-02-07

**Authors:** Tiago Alexandre Cocio, Vivian Caso Coelho, Gilda Maria Barbaro Del Negro, Ingrid Gonçalves Costa Leite, Davi Carvalho Leal Gomes, Roseli Santos de Freitas Xavier, Roberto Martínez, Valdes Roberto Bollela, Gil Benard

**Affiliations:** 1 Universidade de São Paulo, Faculdade de Medicina de Ribeirão Preto, Departamento de Clínica Médica, Ribeirão Preto, São Paulo, Brazil; 2 Universidade de São Paulo, Faculdade de Medicina, Instituto de Medicina Tropical de São Paulo, Laboratório de Investigação Médica em Micologia (LIM-53) São Paulo, São Paulo, Brazil; 3 Universidade de São Paulo, Faculdade de Medicina, Hospital das Clínicas, Laboratório de Investigação Médica em Imunologia (LIM-48), São Paulo, São Paulo, Brazil

**Keywords:** COVID-19, COVID-19-associated pulmonary aspergillosis, Aspergillus welwitschiae

## Abstract

This study reports the first documented case of COVID-19-associated pulmonary aspergillosis (CAPA) caused by *Aspergillus welwitschiae* in the Americas, which occurred in a rural area of Sao Paulo State, Brazil. The case involves a 52-year-old woman with COVID-19, hypertension, and dyslipidemia, who was admitted following severe respiratory deterioration. Imaging tests revealed extensive pulmonary involvement, including nodular and cavitary lesions indicative of CAPA. Bronchoalveolar lavage (BAL) analysis identified *Aspergillus* spp. using morphological and molecular techniques, and sequencing of the *ben*A gene confirmed the isolate as *A. welwitschiae*, closely related to the reference strain CBS 139.54. Matrix-assisted laser desorption/ionization-time of flight mass spectrometry further validated this identification. Traditionally recognized as a plant pathogen, *A. welwitschiae* has recently been implicated in human diseases, such as otomycosis, and is increasingly detected in respiratory samples. However, its association with CAPA remains underreported globally, particularly in the Americas. This case highlights the critical importance of accurate fungal diagnosis, as overlapping morphological features among *Aspergillus* species can hinder clinical management. The identification of *A. welwitschiae* in this context raises concerns about its potential as an emerging pathogen in agricultural regions, where environmental exposure may drive its epidemiological relevance. Given the growing recognition of *A. welwitschiae* as a CAPA agent, this report underscores its importance in the epidemiology of the disease and its potential role in regions with high agricultural activity. Accurate identification is essential for guiding targeted interventions and addressing the public health risks posed by this emerging fungal threat toCOVID-19 patients.

## INTRODUCTION

COVID-19, caused by SARS-CoV-2, affects patients of all ages, sexes, and comorbidities, with primarily pulmonary manifestations, but it also impacts the cardiac, hepatic, endocrine, gastrointestinal, and renal systems^1^. Additionally, the association between COVID-19 and opportunistic fungal infections is still under investigation, particularly regarding the molecular epidemiology of these agents and their response to anti-fungal drugs^1^.

*Aspergillus* spp. has been identified as one of the main etiological agents that cause pulmonary fungal infections in COVID-19 patients, a condition known as COVID-19-associated pulmonary aspergillosis (CAPA)^2^. The pathogenesis of CAPA seems to be focused in the airways, differing from the angio-invasive aspergillosis in neutropenic patients^3^. The presence of *Aspergillus* spp. in the respiratory tract may indicate CAPA but can also result from colonization or contamination, requiring additional investigation for its diagnosis^4,5^.

In CAPA cases reported worldwide, the most prevalent species associated with this disease is *A. fumigatus* ^6^. However, other species, such as *A. flavus, A. niger*, and *A. terreus*, are also implicated in the development of CAPA, depending on the region and environmental conditions^6^. In countries like France and Portugal, there have been reports of species considered “uncommon” in invasive aspergillosis (IA) cases, as the *Aspergillus welwitschiae* ^7,8^. This species, traditionally regarded as a phytopathogen, has demonstrated significant potential to cause aspergillosis in humans^9^. This topic has recently been reviewed in Brazil and the first CAPA case was reported in December 2020, in 36 cases out of 736 COVID-19 patients^2^. Diagnosis in Brazil adheres to the criteria established by Koehler *et al*.^2^, using molecular biology, morphology, and other procedures to identify the *Aspergillus* species, primarily *A. fumigatus* and *A. flavus*, although less common species have also been detected^2^.

This study reports the first case of a patient contaminated by an unusual species in the Southeast region of Brazil, which shows *Aspergillus* biodiversity, a cause of diseases in humans, and serves as a warning about how to conduct treatment for CAPA and other Aspergillosis-associated conditions in Brazil and the Americas.

## CASE REPORT

The patient, a 52-year-old woman from the rural area of Ribeirao Preto municipality, Sao Paulo State, Brazil, was diagnosed with COVID-19 by positive nasopharyngeal swab RT-PCR results, and treated with ceftriaxone, azithromycin, and dexamethasone (6 mg/day), not showing improvement. She was then referred to our hospital in October 2021. She reported a history of hypertension, dyslipidemia, and dyspneA. On admission, her arterial blood gas showed a 7.34 pH, 36.4 mmHg pO2, 74.2 mmHg pCO2, 39.0 mMol/L HCO3, 10.9 mMol/L base excess, and 67.3% O2 saturation. With oxygen flow rate of 1.5L/min supplied by a mask, she maintained a 98% O2 saturation, which dropped to below 70% with movement. Her heart rate was 92/min, blood pressure, 120/70 mmHg, and pulmonary auscultation showed bilateral crackles. A blood smear showed anemia (hemoglobin 10.1g/dL), and normal platelets and white cell counts except for discrete eosinophiliA. Blood glucose was 130 mg/dL, and C-reactive protein was 2.0 mg/dL. Renal and liver function tests were normal, and serological tests for HIV, paracoccidioidomycosis, histoplasmosis, and aspergillosis were negative.

**Figure 1:**
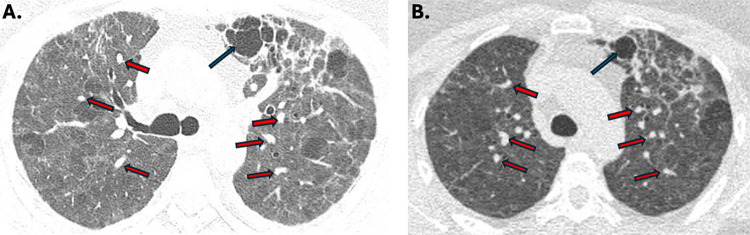
Thorax CT: cavitary lesion (blue arrow) and numerous nodules in lungs. Eighty-eight per cent lung involvement, thickened bronchial walls in the lower lobes, multiple ground-glass opacities, well circumscribed nodular consolidations in the lower lobes, cavitary lesions and areas of emphysema.

A chest CT scan revealed 88% lung involvement, thickened bronchial walls in the lower lobes, multiple ground-glass opacities, well circumscribed nodular consolidations in the lower lobes, cavitary lesions and areas of emphysema (Figure 1). The echocardiogram showed normal heart dimensions and function, except for mild tricuspid valve insufficiency and a possible increase in pulmonary artery systolic pressure.

The patient’s treatment included methylprednisolone (2 mg/kg/day), piperacillin-tazobactam, hydrochlorothiazide, and furosemide, along with her regular medications: atorvastatin, sertraline, and atenolol. She showed progressive respiratory improvement over the 10 days of hospitalization. Methylprednisolone was reduced to 1 mg/kg/day and later replaced by oral prednisone (100 mg/day), with a gradual reduction planned for the following weeks. Upon discharge, she still had cough and some crackles at the lung bases but no longer needed supplemental oxygen, presenting 94% O2 saturation at room air.

During hospitalization, pre-existing interstitial lung disease was considered. Bronchoscopy showed a normal bronchial tree. Bronchoalveolar lavage (BAL) revealed 0.31 cells/µL: 43.5% macrophages, 52.2% lymphocytes, 2.4% neutrophils, and 1.9% eosinophils. Microbiological BAL examination was negative for pyogenic bacteria and acid-fast bacilli but identified *Aspergillus* spp. using morphological methods. Bronchoscopic biopsy showed lung tissue with mild lymphocytic infiltrate, bronchial muscle hypertrophy, and fibroplastic repair. The patient was evaluated approximately five months after hospital discharge. Respiratory symptoms had resolved, she was eupneic with 98% O2 saturation in room air, and lung auscultation was normal. Spirometry results were normal.

### Identification of Aspergillus spp. isolated from the patient

#### Aspergillus spp. strain

The *Aspergillus* spp. was isolated in Sabouraud Dextrose Agar (Oxoid^®^ LTD, Thermo-Fisher Scientific^®^, Basingstoke, Hampshire, England) and supplemented with 0.15 g/L sodium succinate chloramphenicol (Blau Farmaceutica, Florianopolis, Brazil) from a BAL sample collected from the patient.

#### DNA extraction and benA sequencing

The genomic DNA sample (gDNA) from *Aspergillus* spp. strains was obtained as described previously with few modifications^10^. The concentration of gDNA was determined using the NanoDrop 2000^®^ (Thermo-Fisher), and its integrity was verified using a 1% agarose gel diluted in Tris–Acetate-EDTA buffer (1×TAE), a 1 Kb GeneRuler^®^ molecular weight marker (Thermo-Fisher), a GelRed^™^ DNA stain (Biotium, Fremont, CA, United States) as the intercalating agent, and visualized on the UVIDOC photodocumenter (Uvitec, Cambridge, UK). The methodology described by Hubka and Kolarik^11^ was employed to amplify the β-tubulin (*ben*A) (5’ AAT AGG TGC CGC TTT CTG 3’ sense and 5’ AGT TGT CGG GAC GGA AGA G 3’ antisense) of the *Aspergillus* spp. The *Aspergillus* spp. *ben*A PCR reaction was carried out in the Veriti^®^ thermocycler (Applied Biosystems, Foster City, CA, United States) with Platinum^™^ Taq DNA Polymerase enzyme (Thermo Fisher). The final volume of the PCR reaction was 50 μL, containing 50 ng/µL of gDNA and a 2 µM primer concentration of *ben*A. The PCR product was purified using the ExoSAP-IT™ enzyme (Applied Biosystems), following the manufacturer’s instructions. The nucleotide sequence was determined using the 3130XL Genetics Analyzers^®^ sequencer (Applied Biosystems) and the BigDye^®^ Terminator v3.1 Cycle Sequencing Kit (Applied Biosystems) according to the manufacturer’s instructions.

#### Phylogenetics analysis

The *ben*A sequence was analyzed using the BioEdit 7.1^®^ software^12^ and compared with the database using the BLASTn tool^13^. Phylogenetic analysis for *Aspergillus* spp. *ben*A was carried out following the methodology previously described by Cocio *et al*.^14^, using the maximum likelihood (ML) method and the Kimura 2-parameter evolutive model with a 1000 replication bootstrap. The MEGA software (version 11.0, Molecular Evolutionary Genetics Analysis)^15^ was used for phylogenetic reconstruction. The phylogenetic analysis of *ben*A from *Aspergillus* spp. strains was compared to reference strains of cryptic species from the *Nigri* section and two outgroups clades who belong to *Fumigati* and *Flavi*, respectively. Reference strains were: MN969369.1 *Aspergillus welwitschiae* strain CBS 139.54 beta-tubulin (BenA) gene partial cds; EF661089.1 *Aspergillus niger* isolate NRRL 326 beta-tubulin gene partial cds; EF661086.1 *Aspergillus tubingensis* isolate NRRL 4875 beta-tubulin gene partial cds; EF661485.1 *Aspergillus flavus* isolate NRRL 1957 beta-tubulin gene partial cds; EF669791.1 *Aspergillus fumigatus* isolate NRRL 163 beta-tubulin gene partial cds^16^.

#### MALDI – TOF MS identification

*Aspergillus* spp. isolates were identified using the Matrix-assisted laser desorption/ionization-time of flight mass spectrometry (MALDI-TOF MS) method by extracting proteins via the solid Sabouraud Dextrose Agar OxoidTM culture medium method^17^. The Bruker IVD MALDI Biotypertm MicroflexTM LT/SH platform (Bruker Daltonick GmbH & Co. KG, Bremen, Germany) was used at the Hospital das Clinicas da Faculdade de Medicina de Ribeirao Preto, Sao Paulo State, Brazil. The spectra of each *Aspergillus* spp. were evaluated in the Mass Spectrometry Identification (MSI 2.0) data base^18^, using the identification criteria recommended by the software. The *Aspergillus* spp. was evaluated using the MSI 2.0 database, following the species identification criteria provided by the platform: A – species-level identification; B – genus-level identification; C – unidentified, as recommended by the MSI 2.0 platform^18^.

#### Micromorphological analysis of Aspergillus spp.

The micromorphological analysis of the structures of *Aspergillus* spp. was performed by inoculation in potato agar blocks medium (PDA) (Oxoid^®^ LTD, Thermo-Fisher Scientific). These blocks were covered with a sterilized coverslip and incubated at 37 °C for 48h. The coverslips were transferred to a slide containing a drop of lactophenol cotton blue. The material was observed under a bright-field microscopy at 400x magnification, in which hyphae, conidiophores, stipes, vesicles, phialides, and conidia were examined. The morphological analysis of *Aspergillus* spp. was initially performed by observing the micro-morphological characteristics of the fungus and comparing it to the reference strain CBS 122719 - *A. tubingensis* ^16^.

## RESULTS

### Aspergillus spp. identification as A. welwitschiae

**Figure 2:**
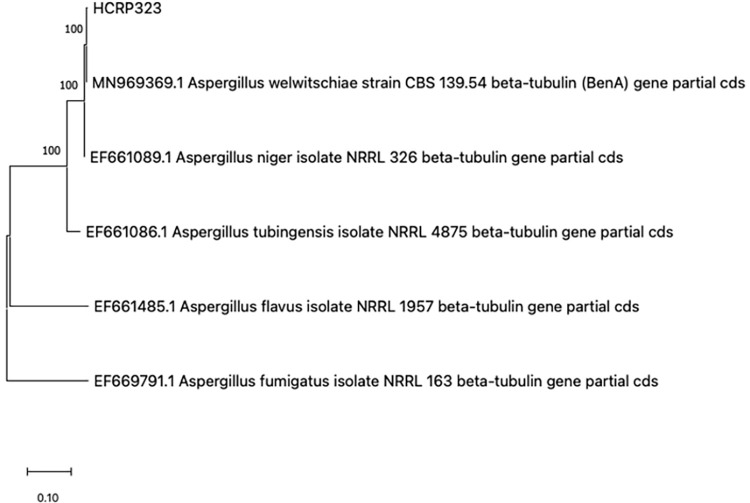
Phylogenetic analysis of *ben*A from *Aspergillus* spp. isolates compared to reference strains of cryptic species – MN969369.1 *Aspergillus welwitschiae* strain CBS 139.54, EF661089.1 *Aspergillus niger* isolate NRRL326, EF661086.1 *Aspergillus tubingensis* isolate NRRL4875, EF661485.1 *Aspergillus flavus* isolate NRRL1957, EF669791.1 *Aspergillus fumigatus* isolate NRRL 163. Evolutionary history was inferred using the maximum likelihood (ML) method based on the Kimura 2-parameter model with bootstrap of 1000.

The *Aspergillus* spp. isolated from the patient, designated HCRP323, had its genomic DnaG amplified via PCR for beta-tubulin (*ben*A), identifying it as *A. welwitschiae* matching 100% with MN969369.1 *A. welwitschiae* CBS 139.54 (Figure 2) in the BLASTN analysis. The phylogenetic tree shows a close genetic relationship with isolate MN969369.1 *A. welwitschiae* CBS 139.5 and genetic divergence from strains of the species *A. niger, A. tubingensis, A. flavus*, and *A. fumigatus*, respectively (Figure 2). This observation confirms that HCRP323 belongs to the *A. welwitschiae* species. The nucleotide sequences of the HCRP323 isolate were deposited in GenBank with the accession number: PQ558662.

In addition to the molecular characterization of HCRP323 using *ben*A sequencing, MALDI–TOF MS was performed and confirmed the clinical isolate as belonging to the species *A. welwitschiae*, with an A score index by the MSI 2.0 spectral database.

### Micromorphological analysis suggests HCRP323 belongs to the Nigri section

**Figure 3:**
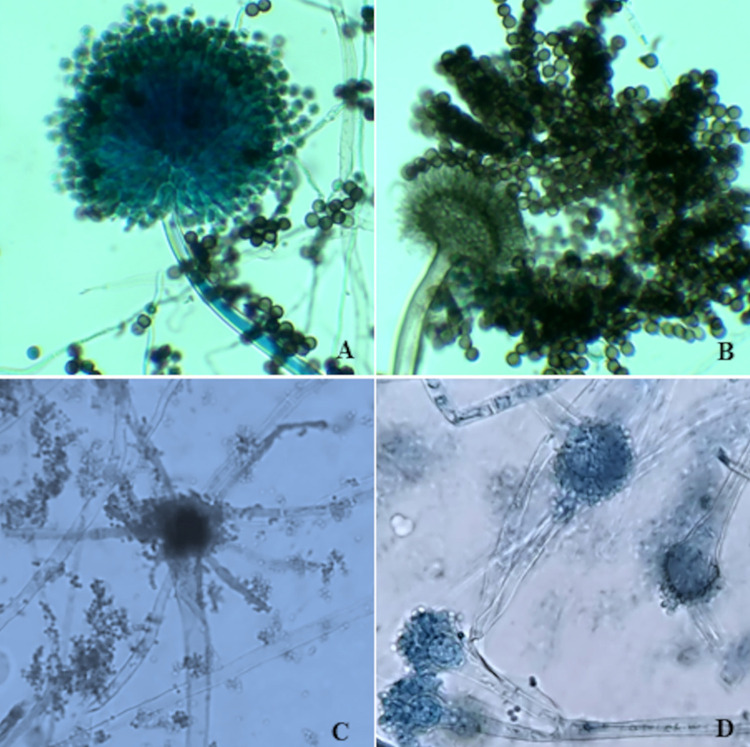
Mycological diagnosis and molecular identification of black *Aspergillus*, section *Nigri* was evaluated using *A. tubingensis* (CBS 122719) as a control. Micromorphology was studied on potato dextrose agar (DIFCO, Becton, Dickinson and Company, France) at 25 °C for three days: in (A) and (B) *Aspergillus tubingensis,* demonstrated presence of globous vesicles completely surrounded by phialides, black conidia; while *Aspergillus welwitschiae* in (C) demonstrated presence of uniseriate conidial heads presented only 2/3 radiated and expanded ampulliform phialides; (D) presence of branching of the conidiophore and uniseriate conidial heads.

Micromorphological analysis compared HCRP323 with reference strain CBS 122719 - *A. tubingensis* and observed vesicles surrounded by phialides, black conidia, and hyaline hyphae, with vesicles measuring 28 μm in diameter and 3 μm smooth-walled microconidia (Figures 3A and 3B). In contrast, HCRP323- *A. welwitschiae* showed 2.8 μm conidia, ranging from hyaline to black, with a smooth to slightly rough surface. Uniseriate conidial heads displayed 2/3 radiation, with some vesicles showing ampulliform phialides under a light microscopy. Vesicles were globose to subglobose, with diameters ranging from small to an average of 20 μm (Figures 3C and 3D). These characteristics are suggestive of the *Nigri* section^16^.

## DISCUSSION

*Aspergillus welwitschiae* (Bres.) Henn. 1907 is a fungal species from the phylum Ascomycota, class Eurotiomycetes, order Eurotiales, family Aspergillaceae, and genus *Aspergillus* ^9^. Previously considered a synonym of the *A. niger* and *A. awamori* species due to its morphological similarities to other black aspergilli, *A. welwitschiae* was described as a new species within the *Nigri* section by Samson *et al*.^16^ by molecular methods (*benA* and *CaM* sequencing). *A. welwitschiae* is considered an opportunistic phytopathogen that naturally inhabits soil, with its spores dispersed through the air, causing diseases in plants like red rot (or trunk rot) in crops like maize, peanuts, grapes, citrus, and sisal in Europe, Africa, Asia, and Brazil^19^. In Brazil, this species is considered an important pathogen in sisal plantations in Bahia State and in the production of citrus, onions, sugarcane, and corn in Sao Paulo State^19,20^. In addition to plant pathogenicity, *A. welwitschiae* is being increasingly reported in patients with acute invasive aspergillosis, chronic aspergillosis, and otomycosis worldwide.

Clinically, *A. welwitschae* (or *A. awamori* in some earlier publications) is an important agent of otomycosis, having been described in European, Middle Eastern and Asian countries^21-23^. More recently, it has been reported as the more frequent species within the *Nigri* section to cause otomycosis in Iran and France, surpassing *A. tubingensis* and *A. niger* ^23,24^, while in China *A. tubigensis* predominates^23^. Furthermore, a recent survey of 70 *Aspergillus* isolates recovered from clinical specimens from a single center in Doham, Qatar, reported identification of *A. welwitschae* in seven of them; all instances (except one) were cases of non-immunosuppressed patients with fungal rhinosinusitis^25^. The remaining case was isolated from the BAL of a patient with *Aspergillus* pneumoniA. Further, *A. welwitschae* has also been reported as an agent of onychomycosis in patients from Hong Kong^26^. Surprisingly, there are no reports of otitis, rhinosinusitis or onychomycosis cases due to *A. welwitschae* in the Americas; nonetheless it has been identified in environment samples in several Latin America countries^27^.

However, recently there have been occasional reports of chronic pulmonary aspergillosis or acute invasive aspergillosis caused by *A. welwitschiae* in patients from Middle Eastern and Asian countries^7,28^. In 2021, with the advent of the COVID-19 pandemic, two cases of *A. welwitschiae* that caused CAPA were described: one in Portugal and the other inFrance^7,8^. Our patient is, to the best of our knowledge, the first report of probable CAPA associated with this species in the Americas.

The COVID-19-associated *A. welwitschiae* infected patient described in France was older (a 74 years-old man) but, as in our case, was immunocompetent. He had a more severe case of COVID-19 than our patient, requiring mechanical ventilation and did not survive despite initiation (5 days) of antifungal therapy. His clinical picture was complicated by a concomitant pulmonary mucormycosis, which characterizes a mixed fungal infection. The patient was defined as a probable aspergillosis case and the risk factors considered were azithromycin administration for 7 days, > 3 weeks high-dose corticosteroids, and the use of mechanical ventilation. On the other hand, the case reported in Portugal was of a 63 years-old immunosuppressed woman (kidney transplantation and methylprednisolone treatment) who required mechanical ventilation. She was also defined as a probable CAPA but received an appropriate course of antifungal treatment (voriconazole, 14 days). She survived^29^.

Several other studies from Europe and Asia investigated the presence of *A. welwitschiae* in a series of mixed clinical samples (but comprising predominantly respiratory samples) collected from immunocompromised patients, patients with prior lung/mucosal diseases who were eventually suspected of aspergillosis, as well as immunocompetent patients^25,30-34^. *A. welwitschiae* was frequently the predominant agent within the *Nigri* section/complex, in general closely followed by *A. tunbingensis*, with *A. niger* and other species more rarely identified. Although this subject requires further investigation, suggestive findings emerged. Takeda *et al*.^32^ showed that *A. welwitschiae* was more frequent than *A. tubingensis* and *A. niger*, but more associated with ear swabs than respiratory samples, while *A. tubingensis* and *A. niger* were more associated with respiratory samples. Studies in which more detailed patients’ clinical data were available point to *A. welwitschiae* being predominantly associated with colonization, while *A. tubingensis* and *A. niger* were more associated with CPA^34^. Remarkably, once again no reports were found on the presence of *A. welwitschiae* in clinical samples from patients in the Americas despite its presence in environmental samples and its association with agriculture diseases. Gits-Muselli *et al*.^33^ pointed to the similarity of the distribution of the cryptic species between the respiratory isolates and the environmental (air) isolates and questioned the causal link of any fungi other than *A. fumigatus* recovered from respiratory specimens on the patients’ symptoms. According to these authors, this link could simply reflect the inefficiency of the pulmonary tract in eliminating fungi, and not an infectious process. In their cohort, of the 99 patients, only 10 were considered possible invasive aspergillosis cases; the remaining 89 had only chronic pulmonary conditions and were not considered to have chronic pulmonary aspergillosis (CPA) despite the isolation of black aspergilli from their respiratory specimens during their follow up. This contrasts with the study by Takeda *et al*.^32^ who considered CPA in 17 of their 43 patients in which black aspergilli were isolated. On the other hand, another issue is that the scarcity of earlier reports of *Nigri* section species in the clinical setting was probably due to the fact that many older studies misidentified other *Nigri* section organisms as *A. niger* based solely on morphological techniques^34^.

According to the 2020 ECMM/ISHAM consensus criteria, our patient is classified as probable CAPA. These criteria are based on the association of (a) patient’s risk factors, which include mainly immunosuppressive conditions, either inherited, secondary (e.g., malignancies), or iatrogenic (chemotherapy or corticosteroid use at an immunosuppressive dose for at least three weeks, which was the case of our patient), (b) clinical factors and (c) mycological evidence^2^. Clinical factors refer to pulmonary lesions suggestive of aspergillosis on pulmonary imaging or bronchoscopy analysis, which in our case were the well circumscribed nodular and cavitary lesions revealed by the pulmonary CT. Finally, the mycological evidence in our patient’s case was the recovery of the *Aspergillus* spp. from bronchoalveolar lavage fluid. Our patient healed favorably from COVID-19 without requiring mechanical ventilation, with the tapering of the medical immunosuppression being apparently sufficient to the recovery of respiratory involvement of the patient^2^. Antifungal susceptibility testing of the isolate was performed following the M38M51S protocol recommended by the Clinical and Laboratory Standards Institute^35^. It presented minimal inhibitory concentrations of all antifungals tested: itraconazole, voriconazole, amphotericin B, and posaconazole (data not shown).

## CONCLUSION

Thus, to our knowledge, we reported the first case of probable CAPA caused by *A. welwitschiae* in the Americas, which occurred in rural Ribeirao Preto, located in the northwest region of Sao Paulo, Brazil. Therefore, we suggest that the emergence of *A. welwitschiae* in this region may be associated with the intense agricultural activity, particularly the cultivation of sugarcane, citrus, and corn.

In addition to highlighting the genotypic frequency of *A. welwitschiae* in areas considered uncommon for this clade, the study suggests that sophisticated advancements in aspergillosis diagnostics should be developed and standardized to assist in identifying so-called “rare” etiological agents. Tools employing molecular methodologies and mass spectrometry can be applied directly to respiratory samples from patients or to cultures obtained from respiratory material isolates. Serological tests also need to be more accurate, utilizing antigens from these species across different regions in a multicenter study involving *Aspergillus* spp. Notably, antigen-antibody tests do not determine species identification.

## References

[1] Abdoli A, Falahi S, Kenarkoohi A (2021). COVID-19-associated opportunistic infections: a snapshot on the current reports. Clin Exp Med.

[2] Koehler P, Bassetti M, Chakrabarti A, Chen SC, Colombo AL, Hoenigl M (2020). Defining and managing COVID-19-associated pulmonary aspergillosis: the 2020 ECMM/ISHAM consensus criteria for research and clinical guidance. Lancet Infect Dis.

[3] Casalini G, Giacomelli A, Galimberti L, Colombo R, Ballone E, Pozza G (2022). Challenges in diagnosing COVID-19-associated pulmonary aspergillosis in critically ill patients: the relationship between case definitions and autoptic datA. J Fungi (Basel).

[4] Ruiz-Ruigómez M, Fernández-Ruiz M, Pérez-Ayala A, Aguado JM (2022). Long-term follow-up of patients diagnosed with COVID-19-associated pulmonary aspergillosis (CAPA). J Fungi (Basel).

[5] O'Shea M, Birkhamshaw E, Khalil R, Wickramasinghe N, Hamad M, Crooks N (2022). Implementation of a diagnostic algorithm for COVID-19-associated pulmonary aspergillosis. J Hosp Infect.

[6] Hoenigl M, Seidel D, Sprute R, Cunha C, Oliverio M, Goldman GH (2022). COVID-19-associated fungal infections. Nat Microbiol.

[7] Benhadid-Brahmi Y, Hamane S, Soyer B, Mebazaa A, Alanio A, Chousterman B (2022). COVID-19-associated mixed mold infection: a case report of aspergillosis and mucormycosis and a literature review. J Mycol Med.

[8] Ranhel D, Ribeiro A, Batista J, Pessanha M, Cristovam E, Duarte A (2021). COVID-19-associated invasive pulmonary aspergillosis in the intensive care unit: a case series in a Portuguese hospital. J Fungi (Basel).

[9] Quintanilha-Peixoto G, Marone MP, Raya FT, José J, Oliveira JA, Fonseca PL (2022). Phylogenomics and gene selection in Aspergillus welwitschiae: possible implications in the pathogenicity in Agave sisalanA. Genomics.

[10] van Burik JA, Schreckhise RW, White TC, Bowden RA, Myerson D (1998). Comparison of six extraction techniques for isolation of DNA from filamentous fungi. Med Mycol.

[11] Hubka V, Kolarik M (2012). ß-tubulin paralogue tubC is frequently misidentified as the benA gene in Aspergillus section Nigri taxonomy: primer specificity testing and taxonomic consequences. PersooniA.

[12] Hall TA (1999). BioEdit A user-friendly biological sequence alignment editor and analysis program for Windows 95/98/NT. Nucleic Acid Symp Ser.

[13] Altschul SF, Gish W, Miller W, Myers EW, Lipman DJ (1990). Basic local alignment search tool. J Mol Biol.

[14] Cocio TA, Nascimento E, von Zeska Kress MR, Bagagli E, Martinez R (2020). Phylogenetic species of Paracoccidioides spp. isolated from clinical and environmental samples in a hyperendemic area of Paracoccidioidomycosis in Southeastern Brazil. J Fungi (Basel).

[15] Tamura K, Stecher G, Kumar S (2021). MEGA11: molecular evolutionary genetics analysis version 11. Mol Biol Evol.

[16] Samson RA, Visagie CM, Houbraken J, Hong SB, Hubka V, Klaassen CH (2014). Phylogeny, identification and nomenclature of the genus Aspergillus. Stud Mycol.

[17] Li Y, Wang H, Zhao YP, Xu YC, Hsueh PR (2017). Evaluation of the Bruker biotyper matrix-assisted laser desorption/ionization time-of-flight mass spectrometry system for identification of aspergillus species directly from growth on solid agar mediA. Front Microbiol.

[18] Normand AC, Blaize M, Imbert S, Packeu A, Becker P, Fekkar A (2021). Identification of molds with matrix-assisted laser desorption ionization-time of flight mass spectrometry: performance of the newly developed MSI-2 application in comparison with the Bruker filamentous fungi database and MSI-1. J Clin Microbiol.

[19] Silva JJ, Bertoldo R, Fungaro MH, Massi FP, Taniwaki MH, Sant'Ana AS (2021). Black aspergilli in Brazilian onions: from field to market. Int J Food Microbiol.

[20] Silva RM, Brito SS, Carmo CO, Soares AC (2019). Controle de Aspergillus welwitschiae e da podridão vermelha com resíduo líquido do desfibramento das folhas de sisal. Cienc Agric.

[21] Szigeti G, Sedaghati E, Mahmoudabadi AZ, Naseri A, Kocsubé S, Vágvölgyi C (2011). Species assignment and antifungal susceptibilities of black aspergilli recovered from otomycosis cases in Iran. Mycoses.

[22] Szigeti G, Kocsubé S, Dóczi I, Bereczki L, Vágvölgyi C, Varga J (2012). Molecular identification and antifungal susceptibilities of black aspergillus isolates from otomycosis cases in Hungary. MycopathologiA.

[23] Zhang L, Wang X, Houbraken J, Mei H, Liao W, Hasimu H (2020). Molecular identification and in vitro antifungal susceptibility of aspergillus isolates recovered from otomycosis patients in Western ChinA. MycopathologiA.

[24] Halvaeezadeh M, Jalaee GA, Fatahinia M, Mahmoudabadi AZ (2023). Aspergillus welwitschiae; an otomycosis predominant agent, new epidemiological and antifungal susceptibility data from Iran. Microb Pathog.

[25] Salah H, Lackner M, Houbraken J, Theelen B, Lass-Flörl C, Boekhout T (2019). The emergence of rare clinical aspergillus species in Qatar: molecular characterization and antifungal susceptibility profiles. Front Microbiol.

[26] Cherif G, Hadrich I, Harrabi M, Kallel A, Fakhfekh N, Messaoud M (2022). Aspergillus flavus genetic structure at a turkey farm. Vet Med Sci.

[27] Sánchez Espinosa KC, Almaguer Chávez M, Duarte-Escalante E, Rojas Flores TI, Frías-De-León MG, Reyes-Montes MD (2021). Phylogenetic identification, diversity, and richness of aspergillus from homes in Havana, CubA. Microorganisms.

[28] Horiuchi H, Watanabe A, Yaguchi T, Ban S, Otsuka T, Miyazaki H (2024). Superficial abdominal surgical site infection caused by Aspergillus welwitschiae: a case report. BMC Infect Dis.

[29] Howard SJ, Harrison E, Bowyer P, Varga J, Denning DW (2011). Cryptic species and azole resistance in the Aspergillus niger complex. Antimicrob Agents Chemother.

[30] Carrara B, Richards R, Imbert S, Morio F, Sasso M, Zahr N (2020). Species distribution and comparison between EUCAST and gradient concentration strips methods for antifungal susceptibility testing of 112 aspergillus section Nigri isolates. Antimicrob Agents Chemother.

[31] Pinto E, Monteiro C, Maia M, Faria MA, Lopes V, Lameiras C (2018). Aspergillus species and antifungals susceptibility in clinical setting in the North of Portugal: cryptic species and emerging azoles resistance in A. fumigatus. Front Microbiol.

[32] Takeda K, Suzuki J, Watanabe A, Narumoto O, Kawashima M, Sasaki Y (2022). Non-fumigatus aspergillus infection associated with a negative aspergillus precipitin test in patients with chronic pulmonary aspergillosis. J Clin Microbiol.

[33] Gits-Muselli M, Hamane S, Verillaud B, Cherpin E, Denis B, Bondeelle L (2021). Different repartition of the cryptic species of black aspergilli according to the anatomical sites in human infections, in a French University hospital. Med Micol.

[34] Samson RA, Hong SB, Frisvad JC (2006). Old and new concepts of species differentiation in aspergillus. Med Mycol.

[35] Clinical and Laboratory Standards Institute (2022). Performance standards for antifungal susceptibility testing of filamentous fungi: supplement M38M51S.

